# Dry resist lamination for wafer-scale fabrication of microfluidic superfusion devices

**DOI:** 10.1038/s41598-025-19744-7

**Published:** 2025-09-25

**Authors:** Rui Liu, Esteban Pedrueza-Villalmanzo, Aldo Jesorka

**Affiliations:** Department of Chemistry and Chemical Engineering, Chalmers University of Technology, 412 96 Göteborg, Sweden

**Keywords:** Microfluidic chips, Open space microfluidics, Multilayer lamination, Dry film photoresist, Engineering, Materials science, Nanoscience and technology

## Abstract

We present a cleanroom fabrication process of free-standing open space microfluidic devices with channel widths of ~ 30 µm on the wafer scale by means of photolithography in combination with lamination technology.

**Supplementary Information:**

The online version contains supplementary material available at 10.1038/s41598-025-19744-7.

## Introduction

Microfluidics in the open space have been widely used in many research areas^1^. For example, probes with the concepts of microfluidics and scanning probes were used for localized staining of cells^2^ and perfusion of brain cells^3^. In 2010, the microfluidic pipette was introduced as a convenient superfusion instrument for single cell pharmacology^4^, lipid film formation^5^, antibody development^6^ and multiple other applications^7–11^. As for the superfusion device, polydimethylsiloxane (PDMS) is used as material, but it is associated with a number of shortcomings. PDMS is incompatible with most organic solvents, e.g. hexane, toluene, and chloroform, which cause swelling and deformation of the elastomer^12^. A few alternative materials that address successfully the solvent compatibility issues exist, but they introduce other problems, such as the sulfur-containing off-stoichiometry thiol–ene (OSTE) polymer^13,14^, and a fluorinated thermoset polymer^15^. The latter involves a dry replica molding process at elevated temperature for fabrication. Mechanical cutting and/or punching are needed to generate channel openings at the end of the fabrication procedure. Moreover, PDMS microfluidic devices have large footprints, which can render positioning cumbersome and cause shadows in the field of view under an inverted microscope. We reported earlier an alternative by using the negative photoresist SU-8^16^ on glass wafers by a bonding process, which was based upon prior microneedle fabrication studies^17,18^. Our approach was promising but also associated with pertaining fabrication difficulties, long resting time for height equilibration, edge beads and separation problems. It has been reported that the edge bead for a 100 µm thick SU-8 exceeds 30 µm^19^. Thus, there still is a need for wafer-scale fabrication methods capable of addressing all or most of these inherent challenges, which we eventually addressed by shifting towards dry negative resist sheets in combination with a lamination process. Dry film photoresist has been thus far mainly used in printed circuit board technology^20^. Due to its processing properties comparable to liquid SU-8, dry films have been used to fabricate millimeter-wave waveguide components^21^, single and multilayer molds^22,23^ and masters for microfluidic devices^24^. We have recently developed and reported a new cleanroom-compatible hard polymer process to fabricate free-standing open space microfluidic devices by stepwise building up devices from multiple layers of commercial SUEX dry film photoresist^25^. This process allows for a direct wafer-scale fabrication without the pre- and post-processing of earlier approaches. The channel width of the reported devices was with 75 µm comparatively large. Typical applications in analytical and diagnostic areas have channels dimensions around 30 µm.

**Fig. 1:**
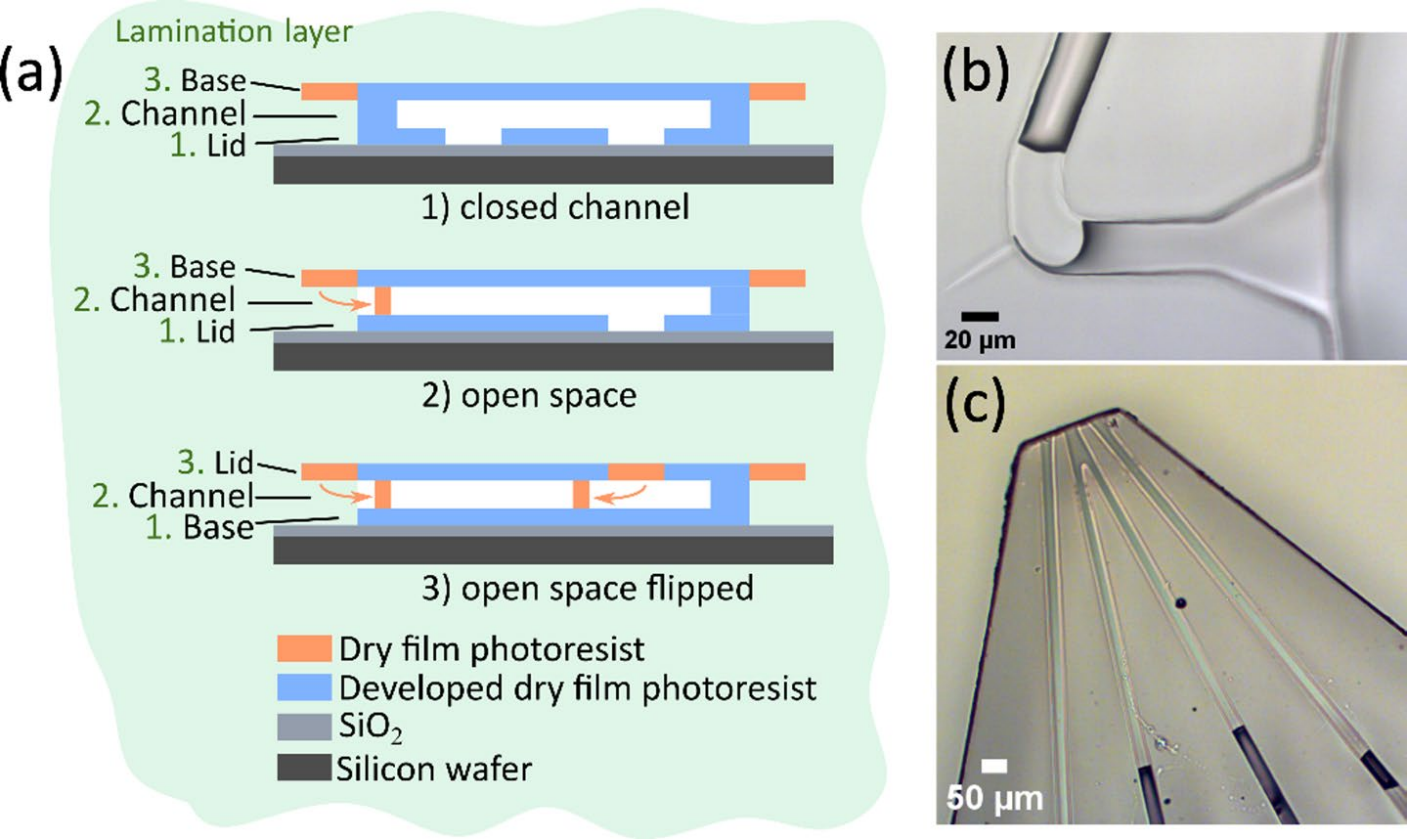
(**a**) Schematic drawing of different microdevice types during the development process. To the left of the chip cross section, the layer lamination order and denomination are shown. (**1**) Closed channel device. The chip inlets (solution access holes) are facing the wafer. (**2**) Open space device. Plug formation in an open space device due to capillary force-driven aspiration of resist/developer mixture into the channel outlets. The chip inlets are unaffected, since they face the wafer. (**3**) Open space device flipped. Similar unfavorable situation with a different layer order, exposing inlets and outlets to the resist/developer mixture. (**b-c**) Optical microscope images of channel blocking according to **a** in an open space microdevice (microneedle) at the inlet (**b**) and the outlet (**c**). The channel width is 30 µm.

When attempting to downscale the channel dimensions to reach this size range, the persistent clogging of the channel inlets and outlets^25^ of the chip led to complete process failure. This particular problem is exclusive to open space devices, where the channel ends are open and get in contact with the developer fluid. In short: during the last development step, the unexposed photoresist in close vicinity to the channel exits is gradually dissolved and reaches a critical viscosity, at which capillary forces draw the relatively thick resist/developer mixture into the channel openings (Fig. 1a). At larger channel dimensions, it is unproblematic to push out these plugs during the development by means of a simple spray bottle, which is commonly used in SU-8 development to free the wafer from resist remnants. However, at smaller channel sizes significantly higher pressure is needed, and the removal becomes challenging and unreliable. The problem is aggravated by the fact that the plugs can occur both on the inlet (solution access holes on the planar chip surface with diameters in the millimeter range, Fig. 1b) and the outlets (Fig. 1c).

Even when attempting to use higher pressure in a layer arrangement that has both channel exits facing the developer (Fig. 1a “open space flipped”), the plug is on one side only pushed deeper into the channel. When attempting to lift off such affected devices in the subsequent process step from common sacrificial materials in an acid bath (buffered oxide etch (BOE) solution for SiO_2_, dilute acid for Cu), the protons rapidly crosslink the plugs and lead to permanently plugged, thus entirely unusable devices. We realized that, in order to fabricate open space devices with typical channel dimensions, a reliable process needed to be devised and implemented.

We present in the following a multilayer lamination process for free-standing open space microdevices with small channel widths of 30 µm, eliminating the experienced channel clogging difficulty. This comprises a simple and efficient approach using photomask exposure on the wafer scale and SiO_2_ as a sacrificial layer. It has the potential to be translated into an industry compatible fabrication process.

We give the essential details regarding device design and fabrication strategy, adhesion problems, wafer reuse, and verification of the device function.

**Fig. 2:**
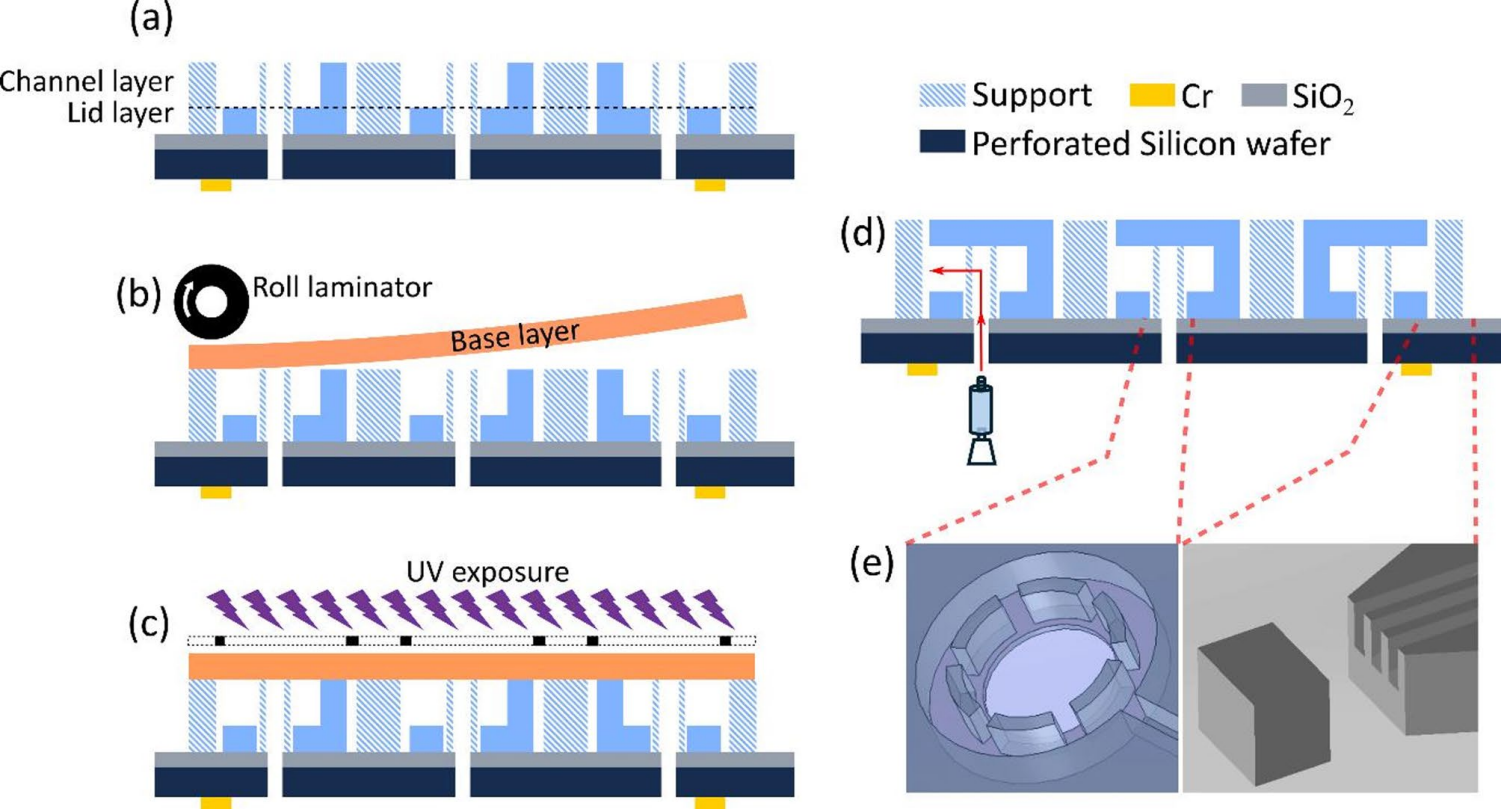
Schematic illustration of the fabrication process of free-standing open space microfluidic device (not to scale). In the cross section views in (**a**–**d)**, the developed SUEX layers are depicted in dark blue, all other structures/materials are specified in the color legend. (**a**) Two developed dry film layers laminated on a perforated silicon wafer with SiO_2_ as a sacrificial layer. (**b**) Laminating the last dry film layer on the developed films. (**c**) Exposing and baking the film using a mask aligner with backside alignment. (**d**) Development of the post-baked film manually by a syringe. (**e**) Magnified regions of the channel ends and close-placed support structures in front of the channels exiting into inlets and the open space.

In order to remove the resist plugs from the channels, a perforated carrier wafer with the orifices aligned to the channel inlets was used in the “open space” layer arrangement (Fig. 2). This enables the application of sufficient pressure through these orifices to the channels during development. Resist plugs can thus be reliably removed from the channel exits. This approach permits wafer-scale microfabrication of free-standing open space microfluidic devices, using a common sulfur- and fluorine-free cleanroom material and a technically straightforward mainstream process.

## Methods

Fabrication of SUEX devices was performed in the Nanofabrication Laboratory at MC2, Chalmers University of Technology. All chemicals and solvents used were of high purity electronic grade, which were provided by the cleanroom facility. A prototype process was developed on the 4″ wafer scale, using double-side polished Si wafers (Ø 4’’, n-type < 100 > , thickness 500 ± 15 µm, MicroChemicals GmbH, Germany). A process flow chart is supplied in Fig. S1.

### Wafer preparation

The wafer was cleaned in acetone for 3 min using an ultrasonicator, quick dump rinse (QDR) treated, and blow dried. The wafer was placed into BOE solution (7:1 ammonium fluoride: hydrofluoric acid) for 5 s, QDR treated, and blow dried. It was thereafter baked at 130 °C for 5 min and cooled down on a metal plate.

### Backside alignment marks

Backside alignment marks are essential to achieve correct alignment of the wafer orifices to the design, and to ensure perfect overlay of the three laminate layers. Two sets of back alignment marks were produced: laser writer exposure was used for the definition of the wafer perforations, which needed on-wafer positions different from the mask aligner, which was used to expose the dry laminate sheets. It is noted that the laser writer may also be used for exposing the dry resist, but the exposure times for the low sensitivity negative resist are with several hours per sheet prohibitively long. Laser writer technology is, however, the optimal choice for the definition of the back alignment marks and the positions of the wafer perforations.

Hexamethyl disilazane (HMDS, Stangl) vapor was applied for 1 min at 100 °C to the cleaned wafer. Microposit S1813 positive photoresist (Shipley) was spin-coated onto the wafer (60 s/4500 rpm/acc 2000), according to the manufacturer-recommended process to get ~ 1.3 µm film thickness, following by a soft baking step (110 °C for 2 min) on a hotplate. Exposure was performed with a laser writer (MLA150, Heidelberg), wavelength 375 nm, dose 170 mJ/cm^2^ and laser defocus level 2. The S1813 was developed in Microposit MF-CD 26 developer (Microresist Technology, Germany) for 1 min, and the wafer was rinsed in water for 1 min, and blow-dried. Oxygen plasma treatment was performed at 50 W for 30 s (BatchTop m/95, Plasmatherm). Thereafter, 65 nm chromium was PVD-deposited on the wafer (Evaporator PVD, Lesker). Note that the chromium layer is 50% thicker than in our previous report, which increases the number of times the carrier wafer can be reused. Negative marks obtained by Si dry etching could be considered as even more robust alternative. A lift-off was performed with REM 400 (Microresist Technology, Germany) overnight. Finally, the wafer was washed with isopropyl alcohol (IPA), QDR-rinsed, and blow dried.

### Perforated silicon wafer

The deep reaction ion etch process (Bosch process) was used to etch perforations into the carrier substrate (*cf.* Fig. S6a). The silicon wafer with backside cross marks was sonicated in acetone for 3 min, QDR-rinsed, and blow dried. The wafer was baked at 130 °C for 5 min and cooled down on a metal plate. HMDS vapor was applied for 1 min at 100 °C to the substrate to improve adhesion between wafer and photoresist. Clariant AZ4562 positive photoresist (Shipley) was spin-coated onto the wafer (60 s/3000 rpm/acc 200), according to the manufacturer recommended process to get ~ 6.2 µm film thickness, following by a soft baking step (110 °C for 6 min) on a hotplate. Exposure was performed using a laser writer, wavelength 375 nm, dose 1000 mJ/cm^2^ and laser defocus level 7. The AZ4562 was developed in Microposit MF-322 developer (Microresist Technology, Germany) for 2.5 min, and the wafer was rinsed in water for 1 min, and blow-dried. The developed wafer was adhered to a 6" carrier wafer by means of viscous diffusion pump oil (SANTOVAC). The SF_6_ dry etch process (Plasma Pro 100, Oxford Instruments) was thereafter utilized to etch holes through the developed wafer, using a standard recipe at a rate of 10 µm/min. The wafers were put into acetone overnight to separate. Finally, the wafer was washed with IPA, QDR-rinsed, and blow dried. An O_2_ Plasma strip was performed at 100 W for 1 min on the perforated wafer to remove photoresist residues.

### SUEX Free-standing open volume device

200 nm SiO_2_ was sputtered onto the top surface of the pre-processed wafer (MS150, FHR Anlagenbau, Germany). The first layer was defined as a lid layer (*cf*. Figures 1a and 2), such that the inlets face the wafer orifices. The coated carrier wafer was O_2_ plasma-treated at 40 W for 1 min before lamination to improve adhesion. 40 µm SUEX was laminated on the wafer with SiO_2_ using a commercial laminator (PRO SERIES 3600, GBC) at 70 °C, speed 5 mm/s. Exposure was performed using a mask aligner (MA/BA 6, Suss MicroTec) with backside alignment, and dose 1350 mJ/cm^2^. Post Exposure Bake (PEB) was performed using a hotplate. The wafer was placed onto the hotplate, covered with a thermally isolating lid and baked while heating from room temperature to 95 °C. Thereafter, the hotplate was turned off to slowly cool the wafer until it reached room temperature, which took approximately 1h.

The second layer was defined as the channel layer. Oxygen plasma treatment to improve adhesion was performed at 40 W for 1 min before lamination. 75 µm thick SUEX was laminated on the wafer at 70 °C, speed 5 mm/s. Exposure was performed using the mask aligner with backside alignment, dose 3000 mJ/cm^2^. PEB was performed in the same way as the previous layer. The two layers of SUEX dry film were developed simultaneously with mr-Dev 600.

The last layer was defined as the bottom layer, sealing the channels except for the exits at the tip of the device. An oxygen plasma step was performed at 40 W for 1 min before lamination. 40 µm SUEX was laminated on the wafer at 65 °C, speed 30 mm/s. Exposure was performed as for layer 1, development and post-treatment was identical to layer 2.

An oxygen plasma descum step was performed at 40 W for 1 min before lift-off, which was performed in BOE for 2 h. SUEX free-standing microdevices were collected, individually rinsed with water, blow dried, transferred into a plastic dish and sealed in antistatic plastic foil under nitrogen. An Olympus MX50 microscope with 5 × and 20 × objectives was used to image the microdevices.

### Holder fabrication and mounting of the microdevice

A holding scaffold was prepared using a 3D resin printer (Formlabs Form3B + , USA), containing 2 on-chip reservoirs and millifluidic (1 × 1 mm) supply channels (*cf*. Fig. S2). Two SMC pressure connectors for 1.2 mm tubing were incorporated in the holder. Because of the photocuring process, the supply channels were designed as close to the bottom of the holder as possible, such that they are exposed late in the process. Around 1 mm produces perfectly open structures. The openings of the holder tip were positioned to match the layout of the microfluidic inlets in the device. The resin holder and a microdevice were manually aligned and interconnected by means of a double-sided 2 mil adhesive transfer tape (3M 467 MPF). The tape was matched with the layout of the device and shaped by a commercial cutting plotter (Cricut Explore 3, USA). The holder was mounted to the microscopy station by means of an electronically controlled micromanipulator (Scientifica, Finland).

### Pressure test

The adhesion quality and tape integrity were determined as described earlier^25^ by applying 2 bars of pressure. The pressure test experiments were conducted on two freshly fabricated devices, having open and sealed channels, respectively. Epoxy glue was used to seal the channel exits. The applied pressure was monitored by a Pascolo EEx digital manometer (Keller AG) and recorded by the device’s software CCS30 (*cf*. Fig. S3).

### Determination of flow rate

**Fig. 3:**
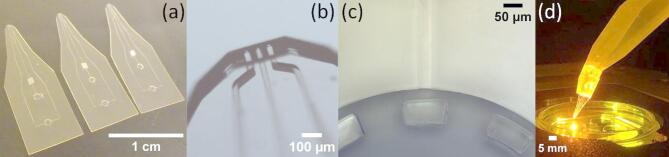
(**a**) Photograph of open space microfluidic devices. (**b**) Optical microscope image of the open tip region of the microdevice. (**c**) Optical microscope image of support pillars located close to the channel inlet after lift-off process. (**d**) Photograph of an assembled setup during a function test experiment of the fabricated microdevice under an inverted microscope.

The fabricated devices are of the most simple configuration and feature three channels, one injection and two adjacent aspiration channels. (*cf*. Figure 3a). The flow rate determination experiments were performed gravimetrically using a series of increasing drive pressure values as reported earlier^25^. Water evaporation loss was considered since the droplet formation time was approximately 40 min at 60 mbar. Evaporation rates were also determined, and the flow rate was corrected accordingly (*cf*. Fig. S5).

### Function testing for microscopy applications

**Fig. 4:**
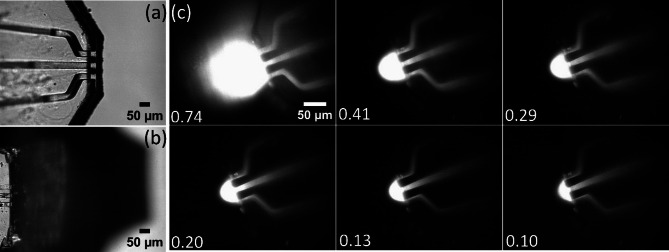
Optical microscope images of the tip of SUEX microdevice (**a**) and PDMS device (**b**) in an open space at 10 × magnification. (**c**) Fluorescence images of the hydrodynamic fluid confinement area at the tip of the microdevice in an open space under different outflow/inflow ratios (denoted in each panel) at 20 × magnification.

The holder with connected microfluidic device was loaded with a Rhodamine B (Sigma Aldrich, Sweden) solution and the wells connected to a multichannel pneumatic air pump via flexible polymer tubing and attached to a micromanipulator. The device tip was positioned inside an open volume, represented by a cell culture dish (Willco Wells, The Netherlands) filled with aqueous buffer, directly above the 20x (numerical aperture = 0.7) objective of an inverted research microscope (DM-IRB, Leica, Germany). The drive pressures were established and adjusted to reach flow settings representative for the different operation conditions shown in Fig. 4c. The confinement area under different outflow/inflow rate *q* in the open space was imaged using a wide field laser-induced fluorescence set-up associated with the microscope. Rhodamine B was excited at λ_ex_ = 546 nm with a diode laser (240mW, Cobolt 06-MLD, Hubner Photonics, Germany), the emitted light was imaged using a Prosilica GX digital camera (Allied Vision Technologies, Germany).

## Results and discussion

### Device fabrication

A lamination process for free-standing open space microfluidic devices with large channel dimensions (75 × 75 µm) were previously reported and applied in a surfactant soap film study^25^. The technical procedure is similar, briefly: two layers of dry film as top lid and channel layer of the microdevice were laminated on a silicon wafer with 200 nm SiO_2_ sacrificial layer beneath, and processed, followed by a third layer to close the structures. At this stage, the original fabrication method consistently failed due to the reduced channel dimensions. With reference to Fig. 1a above, which summarizes the phenomena we faced in this process, open volume and closed channel chips are different in their response to the organic developer. Closed channel devices, if processed with the inlets towards the wafer, have no openings into the developer volume, and are therefore unaffected. The developer does not enter any internal structures. This situation is different for open space devices with channels at the device periphery. While the top layer slowly dissolves in developer, a point in time is reached where the developer-resin mixture is of a viscosity that allows it to be aspired into the channels by capillary forces. The mixture is still comparatively dense, such that a plug is formed that can reach several hundred micrometers into the channels. If this plug is not removed, it will chemically cross-link during the final lift-off step, where an acidic etch solution is applied. If the device is fabricated with its inlets facing the wafer (Fig. 1a), there is no reasonable way to expel the plugs. The alternative, fabricating the device with inlets facing upwards is equally problematic, as any attempt to drive the plugs out by pressurizing the inlets drives the nearest plug even deeper into the chip. We attempted to apply higher pressure, which requires a tight seal around the inlet, only to experience frequent mechanical damage of the epoxy structures. This is in stark contrast to devices with larger channel sizes, where a spray bottle is sufficient to expel the plugs. Therefore, we designed a perforated carrier wafer, produced by deep reactive ion etching by a standard recipe with SF_6_ etchant. (Fig. 2). It allows pressurization through the orifices, which are aligned to the inlets in the devices. The wafer preparation required an additional lithographic step using a thick positive photoresist. A high etching rate of 10 µm/min was applied to reduce etching time, since the uniformity of the perforations is not relevant. Figure 2 shows schematically the process, for brevity starting from the dual-layer laminated wafer (Fig. 2a) and proceeding through lamination of the top layer (Fig. 2b) and exposure (Fig. 2c). The key step is the development (Fig. 2d), where developer is forced through the wafer orifices, expelling the plugs from the tip. Also displayed are the support structures for the resist top layers in front of the channel openings (Fig. 2e).

The results of the fabrication process are displayed in Fig. 3. In panel 3a, examples of multilayer laminate devices are shown, which can be used immediately after production. After lift-off from the wafer by dissolving the sacrificial layer, the devices are floating in the solution bath and have to be retrieved, rinsed and packaged. Due to the very small thickness and transparency, we realized it can be cumbersome to distinguish top from bottom, so it would be advisable to include a direction marker in the design, for example a notch on one side of the structure. Moreover, individual devices could be interconnected by design with thin breakable bridges to allow easier recovery and rinsing after the lift-off step. The photograph in panel 3b shows a magnification of the channel exit area, which reveals well-defined channel geometry and periphery without misalignment. Measurement of the channel dimensions are supplied in Fig. S4. Initial measurement of the dimensions agreed well with the designed features. However, flow rate measurements revealed that the channel dimensions were somewhat smaller than the designed dimensions, as the flow rates did not agree with the calculated values (Fig. S4 and associated tables). We therefore dissected a chip and measured the channel dimensions inside the structure. This analysis showed that the interior channels have been compressed, which led to decreased flow rates. Multiple zones are visible in the cross sections of the dissected chips, suggesting that polymer material is pressed top down into the channels during lamination, essentially adding to the thickness of the top layer. Single channels are otherwise not significantly deformed, the walls remain parallel and at designed distance, except for the very tip region, where the central channel also appears to have been laterally compressed. This is already visible in panel 3b and has been further quantified in Fig. S4a. The lamination parameters are likely of influence in this process, both variations in temperature and roll speed should be further investigated for optimization. However, there are certainly limits, as these parameters influence the interlayer adhesion critically, and cannot be varied over a wide range. The finding that at the channel exits the dimensions are maintained as designed most likely comes from the presence of a large unoccupied air volume in front of the channels, which is preferentially filled with excess, overflowing material during the lamination.

We initially suspected that folding of the top laminate over channel openings on both ends, i.e. on the open volume and inlet side, are responsible for the channel clogging. We therefore designed support structures at 40–100 µm distance from the openings, which kept the top resist sheet from collapsing (Fig. 3c). This alone did not solve the clogging problem but instead positively contributed to retaining the channel shapes and dimensions at the openings.

The chips are remarkable robust and flexible and can be comfortably handled with common round-tipped tweezers without the risk of damage. Sharp tools should be avoided, as point pressure can lead to fractures. The access hole (inlet) diameter of 1 mm is sufficient for manual alignment, even without microscope, to the double-sided transfer tape, which can be equally conveniently attached to the 3D printed holder with integrated sample reservoirs. The performance of this tape attachment, as well as the inter-layer adhesion were tested by means of exposure to 2 bar air pressure, both in open and sealed channel configuration. The tests (*cf*. Fig. S3) revealed that the suggested tape is fully capable of supporting chip operation at the typical one order of magnitude lower pressure range needed to establish operation flow (10–300 mbar), and that the established lamination parameters are appropriate to ensure inter-layer device integrity.

The shape of the tip can in principle be ~ 50% less wide, just sufficiently sized to form a tight seal around the solution access holes on the holder. In this manner close to 40 devices could be on a 4″ wafer (unverified in the laboratory). With respect to wafer yield, all of the structures recovered from a wafer were so far functional, although with small differences in flow performance (*cf.* Fig. S5). There is currently not enough data for exhausting statistics on this matter. Air bubbles can occasionally interfere with channels, either block or interconnect them. The estimated yield for small series at this stage is somewhere between 80 and 100%.

### The following sections discuss a few additional fabrication process and device application details of relevance

#### Inter-layer adhesion

Oxygen plasma treatment was performed before laminating each layer to improve adhesion^21,26^. The first layer lamination speed is important to the adhesion between SUEX dry film and wafer surface. We found that a slower speed is beneficial: at 65 °C, 5 mm/s roll speed led to perfect surface attachment, while at 10 mm/sec, the adhesion was poor, and the film lifted off during the first development. Plasma treatment could not improve this situation. Similar adhesion difficulties were occasionally observed between SUEX layers, when the lamination parameters were not suitable. Air inclusion artifacts at too low roll speed became apparent locally at the channel exit boundaries after the second development step (*cf*. Fig. S6d). If these air inclusions are only regional, they have no influence on the function of the particular flow recirculation device (“microfluidic pipette”^27^) we re-designed, but this could be of concern with other device designs featuring closely spaced channels.

#### Exposure and development

Instead of the laser writer used earlier, photomasks were prepared for each exposure. This is advisable since the time necessary for the mask process is dramatically reduced in comparison to laser writer exposure of the negative epoxy resist. In contrast to several hours on the laser writer, the exposure time of each layer was on the order of a few minutes, e.g. 1.5 min for a 40 µm dry film. Layer alignment using alignment marks on the backside of the wafer was performed prior to each exposure. Back side alignment is particularly important for the topmost layer, since focusing on top alignment marks through the multiple resist layers is challenging and increases the risk of misalignment. PEB did not cause any problems. It is well-known for the conventional SU-8 process that avoiding sharp angular corners in channel design and chip outline prevents stress-related fracture formation during baking steps.

Spray development in which a stream of developer ejected from a spray bottle was applied to microdevices works for cleaning unexposed photoresist from channels > 50 µm^25^. For devices with smaller channel dimensions, we used a syringe with an O-ring seal attached to the outlet and flushed sequentially every single channel manually. This can be done either in the developer bath (wet), or outside (semi-dry) with the wafer facing a cleanroom tissue. The procedure allows to successfully process 4″ wafers without any mechanical damage to the devices, but for larger wafer sizes and many channels/orifices it may become cumbersome. If up-scaling is desired, an automatic development support tool is conceivable and recommended.

#### Wafer reuse

A 65 nm chromium layer was evaporated onto the wafer backside to produce alignment marks. The final BOE lift-off was found to slowly degrade the crosses (*cf*. Fig. S6b), eventually leading to misalignment and resin residue artifacts due to poor definition of the marker shapes (*cf*. Fig. S6c). Since the RIE-treated wafer should be re-used as many times as possible, degradation of the alignment marks reduces this capacity. This issue can be solved by using a different, less affected material for the crosses, or by developing an acid-free lift-off strategy. We are currently continuing process development towards the latter, and also investigate the usability of negative markers, i.e., etched structures.

#### Device application

The fabricated device was tested for microscope applications (Fig. 3d), using a fluorescent dye solution for imaging.  The fluidic performance was found comparable to the respective PDMS device^4^. Figure 4a shows a brightfield image of the laminated device at 10 × magnification under an inverted microscope in comparison to a respective image of a PDMS device (Fig. 4b). The difference in device height is prominent, and the shadow cast by the large PDMS overhead structure is not present with the laminated chip, which is of advantage when positioning the tip at larger magnifications. An apparent difference is the height of the bottom-most layer, which is only 10 µm in the PDMS device^4^. SUEX/ADEX sheets are commercially available down to a thickness of 5 µm, if the channel to surface distance is critical, the fabrication can be adapted. Pressure values for aspiration and injection are within a comparable range  < 300 mbar, therefore the devices can be operated with existing pumps. A recirculation zone was established^4^ and its size was controllable by adjusting the outflow/inflow ratio up to a critical ratio, at which point the confinement was lost (Fig. 4c).

An example of the positioning of the device under an inverted microscope is shown in Fig. 3d, using a 3D printed holder. According to our microscopy application experience, the positioning angle of the open space device often led to practical problems. Translating the holder at low positioning angles, the device body eventually touches the rim of a culture dish, which limits the area inside the dish that can be superfused. At high angles, the condenser of the microscope typically sets a positioning limitation. The small footprint of the laminated devices can greatly improve these issues. Our 3D printed holder features a 45° downward tilt at the tip, allowing for a steep angle between microscope table and chip plane. Moreover, with the combination of laminated tip and 3D-designed holder, the shape of the solution supply reservoirs within the holder structure is rather unproblematic, and the reservoir sizes can be defined over a large volume range. For PDMS devices, a tapered reservoir shape is typically necessary for separation from the mold during fabrication, and the published optimal volume was ~ 35 µl^27^. For our function testing, cylindrical wells with a volume of 140 µl were used. We note that, if the holder bulk is desired to be reduced and the well volume accordingly decreased, the shape of the wells should be optimized such that dead volumes are avoided. The supply channels with dimensions of 1 × 1 mm can be considered as an addition to the supply reservoirs. We note that at this size the channels do not contribute to the flow rates at the much smaller channel outlets. Long supply channels somewhat increase the filling time, i.e. the time the solutions need to reach the channel outlets when readying the system for use. It is also noted that a valuable benefit of the existing PDMS microfluidic pipette is its intended one-time use as a consumable unit. The 3D printed holder is currently not a consumable part, but could be developed into such an option, provided that a cost-efficient design/material combination can be established. Moreover, recent improvements in 3D printing technology have made direct resin printing of channels with dimensions down to 18 × 20 µm feasible^28^. The described process involved development of a suitable resin, which sufficiently limits light penetration and thus out of plane exposure, which is yet to be achieved with current commercial formulations. This opens the possibility to print the microfluidic tip and the solvent reservoir attachment/holder as a monolithic consumable. The main resin material poly(ethylene glycol) diacrylate (PEGDA) has, in contrast to SUEX, only limited resistance to solvents such as acetone and toluene, but would be well suitable for aqueous solutions.

## Conclusion

We present a multilayer dry resist route to reliably fabricate open space microfluidic superfusion devices of small channel sizes on the wafer scale. Fabrication suffered initially from persistent resist plugs at the channel exits. This problem appears to be specific for open volume devices such as microneedles and microfluidic pipettes, and is not of concern for closed channel designs produced by the same fabrication route. We introduced a perforated carrier wafer, which gives access to the channel interior for removal of the plugs during resist development. Application of developer is the key step in the removal of the resist plugs. It is currently accomplished by manually pressurizing the channels through the wafer perforations, larger scale fabrication might require a support tool to facilitate the process. We found distortions of channel geometries after the process, showing that current commercial plastic film laminators are not optimal for resist lamination. The experienced changes in channel dimensions need further systematic characterization for assessing to what extent they can be taken into consideration during device design. Flow rate measurements of larger batches are advisable in this context. Structural and application testing revealed that the laminated microfluidic devices are robust and functional and can with several practical benefits be used under operation conditions established for earlier developed microdevices of comparable function.

## Supplementary Information

Below is the link to the electronic supplementary material.

Supplementary Material 1

Supplementary Material 2

## Data Availability

The data supporting this article has been included in the Supplementary Information.
