# Supplementary material for: Dry resist lamination for wafer-scale fabrication of microfluidic superfusion devices
Source: Sci Rep. 2025 Sep 25;15:32929. doi: 10.1038/s41598-025-19744-7 (PMC12464215; doi:10.1038/s41598-025-19744-7)
Supplement: Supplementary file 1 — Supplementary Material 1 [file 41598_2025_19744_MOESM1_ESM.pdf]

Electronic Supplementary Information

Dry resist lamination for wafer-scale fabrication of microfluidic superfusion devices

Rui Liu, Esteban Pedrueza-Villalmanzo, Aldo Jesorka*

Department of Chemistry and Chemical Engineering, Chalmers University of Technology, Göteborg 412 96, Sweden

***Corresponding author:** aldo.jesorka@gomod.eu

1. Process Flow Chart

Wafer Cleaning

1. Solvent Cleaning
Acetone, Ultrasonicator
3 min

2. Rinse
QDR
3 cycles

3. Drying
Air gun
until dry

4. Native SiO_2 etching
BOE bath
5 s

5. Rinse
QDR
3 cycles

6. Drying
Air gun
until dry

7. Baking
Hotplate
130°C, 5 min

Backside Alignment Marks

8. Pre-treatment
HDMS, hotplate
100°C, 1 min

9. Resist coating
S1813, spin coater
60 s / 4500 rpm / acc 2000

10. Baking
Hotplate
110°C, 2 min

11. Exposure
Laser writer
375 nm, 170 mJ/cm^2, Def. 2

12. Development
MF-CD 26
1 min

13. Rinse
DI-water
1 min

14. Drying
Air gun
until dry

15. Surface activation
O_2 Plasma
50 W, 30 s

16. Metallization
Cr, evaporator
65 nm

17. Lift-off
REM 400
overnight

18. Solvent cleaning
IPA

19. Rinse
QDR
3 cycles

20. Drying
Air gun
until dry

Perforated Carrier Wafer

21. Solvent Cleaning
Acetone,ultrasonicator
3 min

22. Rinse
QDR
3 cycles

23. Drying
Air gun
until dry

24. Baking
Hotplate
130°C, 5 min

25. Pre-treatment
HDMS-hotplate
100°C, 1 min

26. Resist coating
AZ4562, Spin coater
60 s / 3000 rpm / acc 200

27. Baking
Hotplate
110°C, 6 min

28. Exposure
Laser writer
375 nm, 1000 mJ/cm^2, defocus level 7

29. Development
MF-322
2.5 min

30. Rinse
DI-water
1 min

31. Drying
Air gun
until dry

32. DRIE
SF_6 Plasma
10 µm/min

33. Carrier separation
Acetone
overnight

34. Solvent cleaning
IPA

35. Rinse
QDR
3 cycles

36. Drying
Air gun
until dry

37. Plasma cleaning
O_2 Plasma
100 W, 1 min

Device Fabrication

38. Sacrificial layer deposition
SiO_2, Sputter
200 nm

39. Surface activation
O2, Plasma
40 W, 1 min

40. Lamination
Roll laminator
40 µm SUEX, 70°C, 5 mm/s

41. Exposure
Mask aligner
1350 mJ/cm^2

42. Baking
Hotplate
60 min

43. Lamination
Roll laminator
75 µm SUEX, 70°C, 5 mm/s

44. Exposure
Mask aligner
3000 mJ/cm^2

45. Baking
hotplate
60 min

46. Development
mr-Dev 600
10-15 min

47. Surface activation
Plasma
40 W, 1 min

48. Lamination
Roll laminator
40 µm SUEX, 65°C, 30 mm/s

49. Exposure
Mask aligner
1350 mJ/cm^2

50. Baking
hotplate
60 min

51. Development
mr-Dev 600
5 min

52. Plasma cleaning
O2, Plasma
40 W, 1 min

53. Lift-off
BOE
2 h

54. Rinse
DI-water

55. Drying
Air gun
until dry

56. Packaging
N_2, vacuum sealer

Figure S1. Process flow chart for the fabrication of three-layer laminated microfluidic devices.

2. Chip holder

A 3D resin printed holder with two reservoirs was prepared to connect the microdevice with pressure supplier. The downward tilted tip allows the microdevice to be positioned in a near vertical position under an inverted microscope by a micromanipulator. A Ø7 mm steel rod can be fixed to the rear end of the holder for attachment to a micromanipulator. The two cylindrical reservoirs provide a large volume of sample during experiments without the need for reloading and are accessed through SMC Quick-connect fittings for OD 2mm tubing (M3 thread).

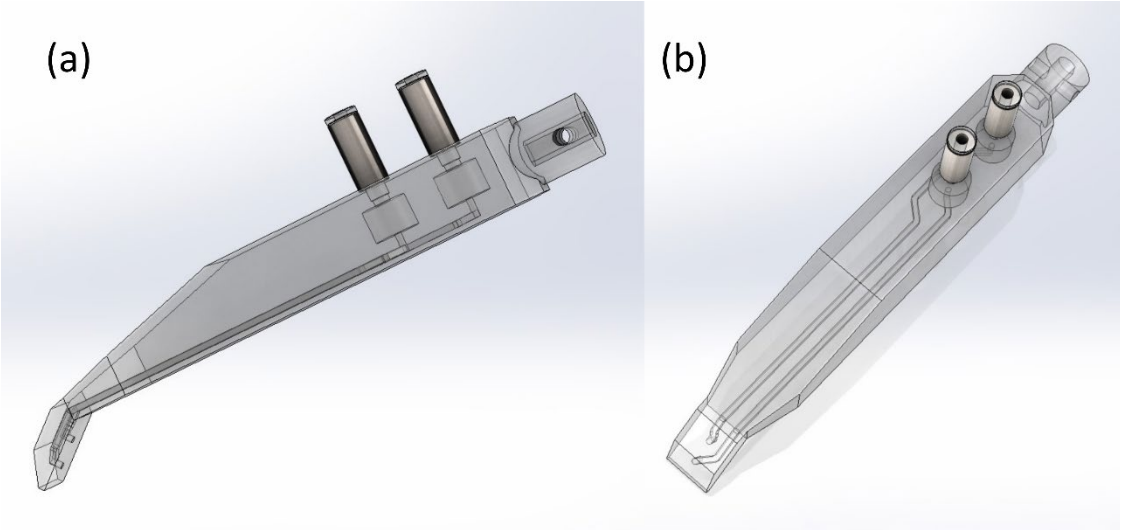

Figure S2. Engineering drawing of the 3D printed resin holder with two cylindrical reservoirs, a 45°downward titled tip and two SMC pressure connectors. **a** and **b** are two different views of the holder.

3. Pressure test on closed and open channels.

The pressure test was conducted on closed and open channels in two freshly fabricated microdevices at 2 bar test pressure. The pressure test gave simultaneous confirmation that a) the interlayer adhesion is sufficient to endure the operation pressure (<300mbar), and b) the tape seal is reliably interconnecting chip and holder. **Fig.S3** shows the results for two test regimes, panel **a** for epoxy-sealed channels, and panel **b** for open channels. 2 bar air pressure was applied and held for 10-20s. Thereafter the supply was closed, and the leaking was monitored. In case of closed channels, the leak rate at 2 bar holding pressure is in all instances ~10 mbar/s, which we attribute to the unoptimized pressure connectors.

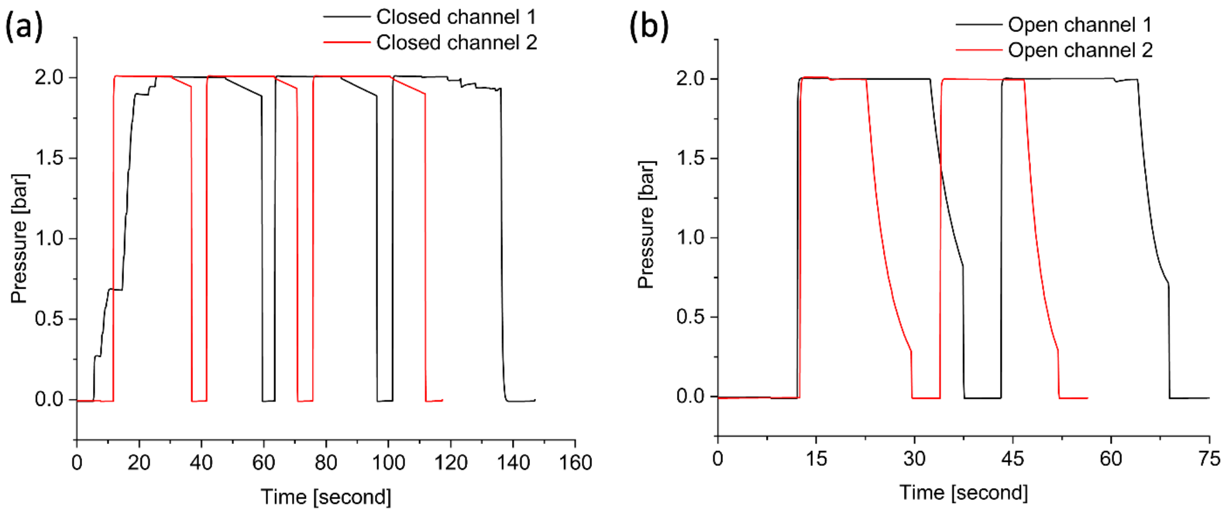

Figure S3. Pressure test conducted on closed channels (**a**) and open channels (**b**) in two freshly fabricated microdevices.

4. Channel dimensions and flow rate measurements

The dimensions of the channel cross sections of the laminated devices were measured with a microscope camera in comparison with the designed dimensions. **Fig.S4** shows the analyzed images with the measurements included as overlays. Panel **a** shows the dimensions for the channel's outlets at the device tip. Panel **b** shows the analysis results for a channel inside the chip structure, obtained by a blade cut perpendicular to the main axis approximately 5 mm from the tip. Panel **c** shows a region on the periphery of the chip parallel to the long axis, revealing the interlayer material structure of the multilayer laminate. The views of all three images are approximately equally magnified for direct comparison.

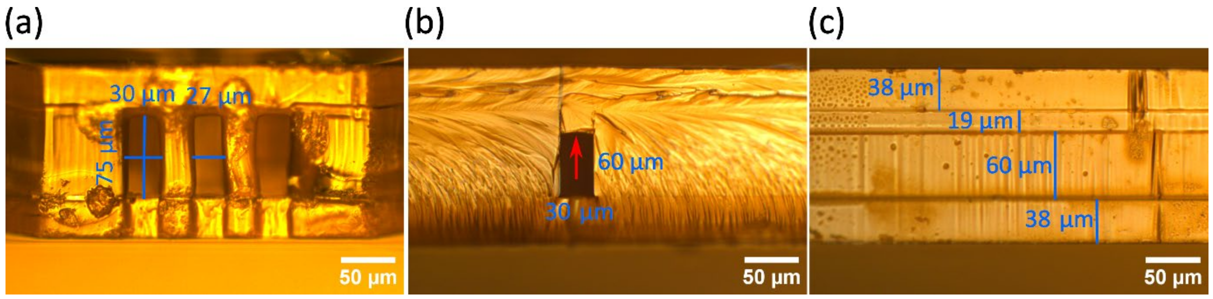

Figure S4. Optical microscope images of open tip into the open space (**a**), channel section with the red arrow pointing to the contact inference between two layers (**b**), side view of the frame (**c**) of a microdevice. Blue solid lines represent measured dimensions.

The flow conductance values *G* were determined for the given rectangular cross-section from the channel dimensions, where *Q* is the measured flow rate, Δp is the supply pressure, *w* is the channel width, h is the channel height, η is the fluid viscosity, and *L* is the channel length:

$$G = \frac{Q}{\Delta p} = \frac{1}{R_{hyd}} \approx \frac{w^3 h}{12\eta L}\left[1 - 0.63\frac{w}{h}\right] h > w$$

In our microdevice, the channel height *h* is larger than its width *w*. The tables **S1** and **S2** give the details for the two channels in the device. The channels were dimensioned such that the operation pressures were in a practical range for a pneumatic pump. This required designing a loop for length extension of one of the channels, such that both are of approximately equal length.

The flow rates were determined gravimetrically[1], at long fluid collection times the measurements were corrected for evaporation loss.

Table S1. Flow rate vs pressure determination by different methods for channel 1.

	Calculated from design	Measured	Calculated from **Fig.S4b**
Channel dimensions ($w \times h \times$ L/µm)	75 x 30 x 23074	-	60*30*23074
Conductance (nL s^{-1} $mbar^{-1}$)	0.54704	0.30959	0.40078

Table S2. Flow rate vs pressure determination by different methods for channel 2.

	Calculated from design	Measured	Calculated from **Fig.S4b**
Channel dimensions ($w \times h \times$ L/µm)	75 x 30 x 20086	-	60*30*20086
Conductance (nL s^{-1} $mbar^{-1}$)	0.62842	0.3832	0.46040

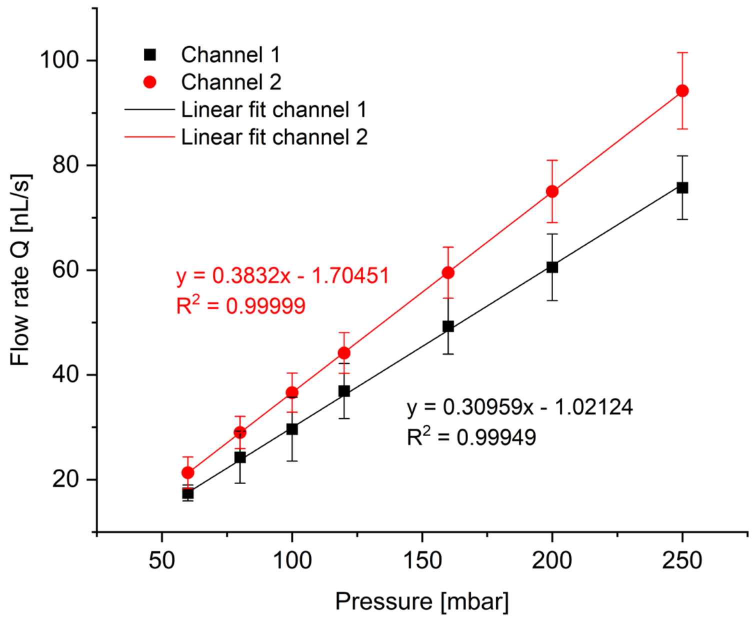

Figure S5. Gravimetric flow rate determination for all channels in the open space microfluidic device under different supply pressure.

4. Microdevice fabrication

Fig.S6a shows a region of a silicon wafer with Ø 0.8 mm perforations used in the microdevice fabrication process. **Fig.S6b-d** are related to difficulties experienced in the fabrication procedure. After reusing the perforated wafer a few times, the chromium alignment marks were successively eroded during the BOE lift-off steps (**b**) and resulted in misalignment of the chip layers during exposure and development artifacts (**c**). With suboptimal lamination speed and temperature during the final lamination step, the interface between channel and bottom layer showed locally poor adhesion (**d**).

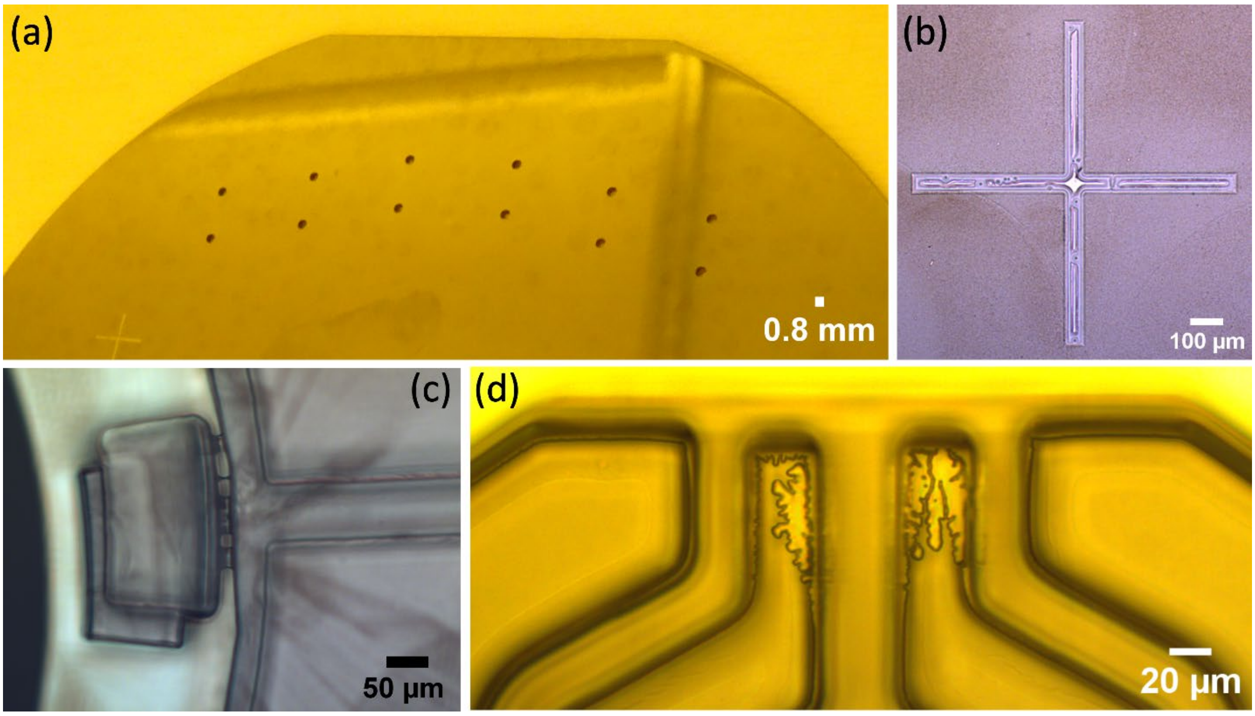

Figure S6. a. Photograph of the backside of a RIE-perforated silicon carrier wafer with one alignment mark visible. The diameter of the openings is 0.8 mm. **b.** Corroded alignment mark after four successive lift-off cycles. **c.** Compromised interlayer adhesion observed in the interface between layers two and three (channel and final layers) of the microdevice. **d.** Optical microscope image of misalignment and undesired overexposure artefacts due to the poor visibility of the corroded alignment marks.

5. Supplementary video

Video S1: Microscopy experiment with a laminated microdevice demonstrating the change of the hydrodynamically confined volume by variation of drive pressures to establish different flow rate ratios. The images in **Fig.4c** of the main manuscript were extracted from this video.

References

1 Liu, R., Pedrueza-Villalmanzo, E., Fatima, F. & Jesorka, A. Free-standing open space microfluidic devices by dry resist lamination. *Microfluid. Nanofluid.* **29**, 1-15 (2025).
